# Text Messaging for Exercise Promotion in Older Adults From an Upper-Middle-Income Country: Randomized Controlled Trial

**DOI:** 10.2196/jmir.5235

**Published:** 2016-01-07

**Authors:** Andre Matthias Müller, Selina Khoo, Tony Morris

**Affiliations:** ^1^ Sports Centre University of Malaya Kuala Lumpur Malaysia; ^2^ College of Sport and Exercise Science Victoria University Melbourne Australia

**Keywords:** exercise, text message, mobile phone, older adults, mHealth, Asia, health behavior, behavior maintenance, physical activity, Malaysia

## Abstract

**Background:**

Mobile technology to promote exercise is effective; however, most evidence is from studies of younger groups in high-income countries. Investigating if short message service (SMS) texting can affect exercise participation in older adults from an upper-middle-income country is important considering the proliferation of mobile phones in developing regions and the increased interest of older adults in using mobile phones.

**Objective:**

The main objective was to examine the short- and long-term effects of SMS text messaging on exercise frequency in older adults. Secondary objectives were to investigate how SMS text messages impact study participants’ exercise frequency and the effects of the intervention on secondary outcomes.

**Methods:**

The Malaysian Physical Activity for Health Study (_my_PAtHS) was a 24-week, 2-arm, parallel randomized controlled trial conducted in urban Malaysia. Participants were recruited via health talks in resident associations and religious facilities. Older Malaysians (aged 55-70 years) who used mobile phones and did not exercise regularly were eligible to participate in the study. Participants randomly allocated to the SMS texting arm received an exercise booklet and 5 weekly SMS text messages over 12 weeks. The content of the SMS text messages was derived from effective behavior change techniques. The non-SMS texting arm participants received only the exercise booklet. Home visits were conducted to collect outcome data: (1) exercise frequency at 12 and 24 weeks, (2) secondary outcome data (exercise self-efficacy, physical activity–related energy expenditure, sitting time, body mass index, grip and leg strength) at baseline and at 12 and 24 weeks. Intention-to-treat procedures were applied for data analysis. Semistructured interviews focusing primarily on the SMS text messages and their impact on exercise frequency were conducted at weeks 12 and 24.

**Results:**

In total, 43 participants were randomized into the SMS texting arm (n=22) and the non-SMS texting arm (n=21). Study-unrelated injuries forced 4 participants to discontinue after a few weeks (they were not included in any analyses). Overall retention was 86% (37/43). After 12 weeks, SMS texting arm participants exercised significantly more than non-SMS texting arm participants (mean difference 1.21 times, bias-corrected and accelerated bootstrap [BCa] 95% CI 0.18-2.24). Interview analysis revealed that the SMS text messages positively influenced SMS texting arm participants who experienced exercise barriers. They described the SMS text messages as being encouraging, a push, and a reminder. After 24 weeks, there was no significant difference between the research arms (mean difference 0.74, BCa 95% CI –0.30 to 1.76). There were no significant effects for secondary outcomes.

**Conclusions:**

This study provides evidence that SMS text messaging is effective in promoting exercise in older adults from an upper-middle-income country. Although the effects were not maintained when SMS text messaging ceased, the results are promising and warrant more research on behavioral mobile health interventions in other regions.

**Trial Registration:**

Clinicaltrials.gov NCT02123342; http://clinicaltrials.gov/ct2/show/NCT02123342 (Archived by WebCite at http://www.webcitation.org/6eGSsu2EI).

## Introduction

Population aging is a global phenomenon that is projected to continue [1,2]. In 2013, there were approximately 841 million people globally who were age 60 years or older (11.7%). This figure is expected to grow to more than 2 billion (21.1%) by 2050 [3]. Accelerated population aging takes place in places other than high-income countries (HICs) where more than 80% of the global older adult population will reside by 2050 [3]. Malaysia is an upper-middle-income country that shows rapid population aging [4,5]. The proportion of older adults in the total population is projected to almost double from 2015 (5.8%) to 2040 (11.4%) [6].

The global rise in the older adult population is linked to an increase in the prevalence of noncommunicable diseases (NCDs), disability, and other health problems which, in turn, increase the burden on public and private health care systems [1,2,7,8]. This is especially so in non-HICs where the increase of NCDs is far greater than in HICs [2]. Lifestyle factors, especially physical activity (PA) and particularly structured exercise, are commonly put forward as essential determinants of good physiological and psychological health in older age [2,9-11]. For example, exercising older adults enjoy increased brain plasticity and cognitive function [12], reduced risk of cardiovascular diseases [13], and reduced metabolic risks [14]. Additionally, older adults who start following a regular strength-training regimen are likely to reduce body fat and greatly increase muscular strength [13,15]. Despite these benefits, many older adults are not exercising enough [11,16]. A recent review of PA levels in older adults reported that between 0% and 17.2% of older adults are active for 150 minutes per week when measured objectively [16]. Because exercise is a subcategory of PA, exercise levels are even lower. Studies reporting on PA and exercise levels of older adults in non-HICs are rare [17]. However, it seems that older adults in non-HICs, such as Malaysia, are increasingly inactive [18-20]. This was confirmed in a recent study reporting that 88% of older Malaysians are not sufficiently active [20]. The unprecedented increase in the older adult population, especially in non-HICs, imposes a great burden on health care systems. Because PA and specifically exercise are essential for good health in older age, there is a need for innovative and cost-effective interventions to increase exercise levels.

Behavioral health interventions focusing on PA and/or exercise are increasingly delivered via mHealth approaches [21], particularly mobile phones [22,23]. However, most interventions are conducted with younger age groups in HICs [21,24,25]. This is surprising because mobile phone penetration is increasing rapidly in non-HICs primarily because of device affordability [26]. This is evident in the Asia-Pacific region where approximately 3.6 billion people own a mobile phone. This figure is likely to increase in coming years [27]. Mobile phone subscriptions are also increasing in older adults. A study of older adults in the United States showed that 75.9% of adults aged 65 and older own a mobile phone [28]. Research also indicated the willingness of older adults to use mobile phone features that provide beneficial information to them, especially for health [29,30]. Short message service (SMS) text messaging is particularly popular among older adults and is the most frequently used mobile phone feature because little technological expertise is required for sending and receiving SMS text messages [28,31]. Therefore, examining SMS text messaging to promote exercise in older adults residing in an upper-middle-income country (Malaysia) would be informative.

### Prior Research

Text messaging has been shown to be successful in promoting PA and/or exercise in young adults [32], postnatal women [33,34], working women [35], and sedentary women [36] from HICs. Text messaging was either a stand-alone intervention or it constituted a major part within a multicomponent intervention. In contrast, in non-HICs, SMS text-messaging interventions were primarily implemented to address maternal, child, and sexual health as well as disease management [26,37-40]. To our knowledge, only one study addressed exercise using SMS text messages in a non-HIC (India). A lifestyle intervention primarily delivered via SMS text messages to prevent the onset of type 2 diabetes in men was not successful in increasing overall activity levels [41].

Only one study reported on the effects of SMS text messaging in older adults [42]. The authors recruited a small sample of older African-Americans in a 6-week SMS text-messaging trial and found that step counts and leisure-time PA increased significantly. The aim of our study is to examine if SMS text messaging can successfully impact exercise behavior in older adults residing in a non-HIC (Malaysia). Further, we aim to determine if the effects of SMS text messaging on exercise behavior are maintained when the SMS text messages are removed.

### Current Intervention

The Malaysian Physical Activity for Health Study (_my_PAtHS) is a randomized controlled trial (RCT) for older Malaysians who do not follow a regular exercise routine. We chose this age group because population aging is a great challenge for Malaysia [5] and the majority of older Malaysians do not exercise enough [20]. The tropical, hot, and humid climate and safety concerns prevent many older Malaysians from exercising outside (eg, brisk walking). Additionally, a lack of appropriate exercise facilities for older adults and insufficient knowledge on how to exercise contribute to low exercise levels in this age group. Consequently, we introduced specific exercises to our research participants and provided a printed home-based exercise booklet. Text messages served as an encouragement and reminder to follow the exercises. The majority of older Malaysians use a mobile phone (analog or smartphone) and are familiar with the SMS text-messaging feature [43].

Our study aimed to (1) determine if older Malaysians receiving an exercise booklet and weekly SMS text messages exercise more than participants who only receive an exercise booklet, (2) examine if the effects of the SMS text messages are maintained when the SMS text messages are removed, (3), investigate how the SMS text messages support participants to exercise, and (4) investigate the effects of the SMS text messages on secondary outcomes (eg, exercise self-efficacy, weekly PA-related energy expenditure, daily sitting time, body mass index [BMI], grip strength, and lower body strength).

## Methods

### Study Design: Overview

The _my_PAtHS is a RCT that uses a parallel study design. All participants were introduced to a set of exercises and received an exercise booklet. Participants randomized into the SMS text-messaging arm (SMS texting arm) received an additional 60 text messages over 12 weeks. Participants randomized into the other arm did not receive SMS text messages (non-SMS texting arm). After enrollment, the primary study outcome was assessed at weeks 12 and 24. The study design and protocol were approved by the Faculty of Medicine Ethics Committee, University of Malaya, and was registered (Clinicaltrials.gov NCT02123342). This trial is reported according to the CONSORT statement [44] and the CONSORT-EHEALTH extension [45] (Multimedia Appendix 1).

### Settings

The study took place in urban Malaysia (Kuala Lumpur and Petaling Jaya) from June 2014 to January 2015. In Malaysia, 73% of the population lives in urban areas and Kuala Lumpur and Petaling Jaya are the most densely populated cities [46].

### Eligibility Criteria for Participants

Eligible participants were English-speaking community-dwelling Malaysians aged between 55 and 70 years, who were not exercising regularly (no structured exercise more than once weekly), had no health conditions that would restrict moderate exercise, used a mobile phone with SMS text-messaging function, and were interested in health-promoting exercise.

### Recruitment and Enrollment

Participants were recruited from local resident associations and religious facilities in April and May 2014. With the support of representatives from the respective organizations, one study team member conducted health talks for older adults within the recruitment area. The study was briefly introduced as an exercise for health program and eligibility criteria were described (SMS text messaging was not mentioned). Those who were interested in taking part were given an information sheet and asked to provide contact details so that a study team member could call them later. Approximately one week after the health talks, potential participants were called. During this call, eligibility criteria were checked, initial oral consent was obtained, and a baseline home visit was scheduled. Home visits were conducted because some participants did not have personal transportation and the public transportation system is not easily accessible. During the home visits, final eligibility checks were conducted, study procedures were explained (eg, time lines, potential risks), informed consent was obtained, and enrollment finalized.

### Randomization and Allocation Concealment

The overall sample was stratified into participants enrolling with their spouse and participants enrolling without a spouse. There is evidence that older adults enrolling in an exercise intervention with a spouse exercise significantly more than those who do not [47]. Participants in the with-spouse and without-spouse strata were randomized separately on the day of enrollment after baseline measurements were taken and informed consent was obtained. Within strata, restricted randomization into the SMS texting and the non-SMS texting arm was applied to achieve balanced sample sizes. Sealed opaque envelopes with chits indicating the study arm were prepared by a study team member. A different set of envelopes was prepared for individual participants and participants enrolling with their spouse (stratification procedure). The envelopes were shuffled and participants were asked to randomly select one of the envelopes (as a means of allocation concealment). Participants and investigators were not blinded to arms assignment; however, participants were not aware that there were 2 research arms.

### Intervention

During the baseline home visit, all participants were introduced to a set of exercises and received an exercise booklet (_my_PAtHS booklet) developed by one of the study team members, an exercise physiologist with experience in training older adults. This booklet contained information on the benefits of exercise, some safety instructions, and descriptions of 12 age-appropriate strengthening exercises that could be executed without any specific equipment. Brief warm-up and cool-down sections were included as well. The exercises targeted major muscle groups of the arms/shoulders, upper trunk/neck, and legs. They were described using pictures, explanations of key movements, and hints where the exercises should be felt. One practical exercise session was conducted during the initial home visit to ensure correct execution. Participants were advised to exercise as often as possible each week to increase health benefits, but no other formal recommendations were provided. To ensure that participants’ mobile phones were operational and participants were competent using the SMS text message function, they were asked to confirm receipt of a text message sent before the baseline home visit.

During the 12 weeks following the baseline home visit, 60 SMS text messages were sent to SMS texting arm participants (during weekdays). Text messages were sent automatically via an online tool specifically developed for this study. This tool allowed the research team to schedule the SMS text messages for every participant and it was also used to confirm delivery of the SMS text messages (Multimedia Appendix 2). Text messages were scheduled for morning hours between 8 am and 11 am according to participant preference. Text messages were developed from previous research that identified behavior change techniques (BCTs) most successful for increasing exercise self-efficacy and exercise behavior [48,49]. These BCTs were providing instructions to exercise and providing rewards/praise for efforts toward exercise behavior [49]. Text messages contained an instruction to exercise using the _my_PAtHS exercise booklet and a statement that praised the participants’ engagement (Textbox 1). Several unique messages were developed and participants received a different message every day. Participants were not required to reply to SMS text messages because this has been shown to not significantly increase effectiveness [50]. Text messages ceased after 12 weeks. For the non-SMS texting arm participants, all procedures were the same, except they did not receive SMS text messages during the 12-week period.

**Example of a SMS text message sent to SMS texting arm participants.**

**Tailoring:** Hello Mr. Wong, I hope you are well.

**Instruction:** Please do the _my_PAtHS exercises regularly.

**Praise/Reward:** All your efforts will impact your health.

**Closing:** Have fun!

### Outcome Measures

In this study, a mixed methods approach was applied to collect outcome data. Quantitative data was supplemented by qualitative data from semistructured interviews.

#### Quantitative Data

The primary study outcome was weekly exercise frequency (exercise sessions using the exercise booklet). It was assessed immediately after the 12-week SMS texting intervention period and after 24 weeks. This outcome was measured with an exercise log appended to the exercise booklet. Participants were asked to record dates, times, and duration of exercise sessions. During the baseline home visit, participants were shown how to record their exercise routine and one trial was conducted to ensure correct data entry. Additionally, one example of a correct entry was provided (in the booklet). Completed logs were exchanged for new ones on subsequent follow-up home visits.

A number of secondary outcomes were assessed at baseline and at weeks 12 and 24. Exercise self-efficacy is strongly associated with exercise participation in older adults [51] and was a covariate controlled for in this study. Participants were assessed using the Exercise Self-Efficacy Scale (EXSE) [52]. Validity and reliability of the EXSE were established in studies with older adults [53,54]. Participants were asked to rate the level of confidence they had for exercising with the exercise program over the coming weeks (from week 1 to week 8). Response options ranged from 0% (not at all confident) to 100% (highly confident) with 10% increments. The mean was used for analysis.

Physical activity-related energy expenditure, in weekly Metabolic Equivalent of Task (MET) minutes (MET-minutes), and daily time spent sitting (in hours) were measured using the short form of the International Physical Activity Questionnaire (IPAQ). The validity and reliability of this instrument are well established and it is widely used [55]. The IPAQ short form consists of 7 items and was interviewer-administered in this study. Respondents were required to indicate frequency (days per week) and duration (hours and minutes per day) of vigorous PA, moderate PA, and walking during the previous week. Another item asked about the time spent sitting on a normal weekday.

In addition, BMI (in kg/m^2^) was calculated from body height and weight using the Seca Clara 803 Digital Personal Scale (Seca GmbH & Co KG, Hamburg, Germany). We assessed maximum grip strength (in kg force) of the dominant hand using the North Coast Hydraulic Hand Dynamometer (North Coast Medical Inc, Morgan Hill, CA, USA). This device has been used in previous studies with different groups of older adults and provided valid and reliable data [56-58]. On a verbal cue (“ready, go”), participants were instructed to squeeze the device as strongly as possible. The test was performed in the standing position and the mean of 3 trials was used for analysis. Lower body strength was assessed with the 30-second chair-stand test. This valid and reliable test was specifically developed for studies with older adults and has been widely used [59-61]. The number of stands from a chair that can be completed in 30 seconds is the test score.

#### Qualitative Data

During the follow-up home visits, we also conducted semistructured interviews with all research participants to complement the quantitative data. The interviews lasted approximately 20 minutes. We were primarily interested in how the participants in the SMS texting arm perceived the SMS text messages. Questions about the impact of the SMS text messages, their content, and what was done with them were discussed (Multimedia Appendix 3).

### Sample Size Calculation

A total of 36 participants (18 per arm) was estimated to provide 80% power at α=.05 to detect a difference of one weekly exercise session between the arms at week 12, assuming a standard deviation of 1.1 session. We anticipated a dropout rate of 15%; hence, we aimed to include 42 participants (21 per arm) [34,42].

### Statistical Analysis

Statistical analysis using SPSS version 21.0 included descriptive statistics of age, sex, education, employment status, health status, and marital status. The intention-to-treat principle framed the analyses. However, intervention-unrelated injuries resulted in dropouts at week 12 and no primary outcome data was collected from these participants (n=4), consequently invalidating the use of imputation procedures. None of these participants were included in any of the analyses. For all other analyses, we used the last observation carried forward procedure for missing data. We also conducted a per protocol analysis for those participants with complete outcome data using the same procedures as in the intention-to-treat analysis (Multimedia Appendix 4).

Weekly exercise frequency at week 12 (SMS texting period) was compared between study arms using an independent *t* test. Additionally, an analysis of covariance (ANCOVA) was conducted to adjust for the effect of exercise self-efficacy at baseline (covariate) as a key predictor of exercise participation in older adults [54]. For the analysis of weekly exercise frequency at week 24 (analysis of outcome after removal of SMS text messages), mixed between-within subjects 2×2 (time×group) ANOVAs were conducted. Interaction effects were followed up with simple effects analysis. In addition, an independent *t* test was conducted to compare weekly exercise frequency at week 24.

For the secondary outcomes, data were converted into 2 change variables: one between baseline and week 12 and one between baseline and week 24. For each variable, an ANCOVA comparing the change scores between the arms at each time point was conducted with the baseline scores entered as a covariate. For each arm, we estimated model-adjusted means, 95% confidence intervals, and *P* values.

### Interview Analysis

The first author transcribed (not verbatim) the interviews and categorized responses into broad predefined themes (eg, exercise program, SMS text message content, effects of the SMS text messages). Themes were further divided into subthemes that were partly derived from the responses of the participants (eg, exercise benefits, perception of how the SMS text messages affected exercise). Direct quotations from participants were extracted to exemplify the results derived from the interviews. Finally, a coauthor checked the interview analysis results for accuracy and discrepancies were resolved via discussion.

## Results

### Overview

**Figure 1:**
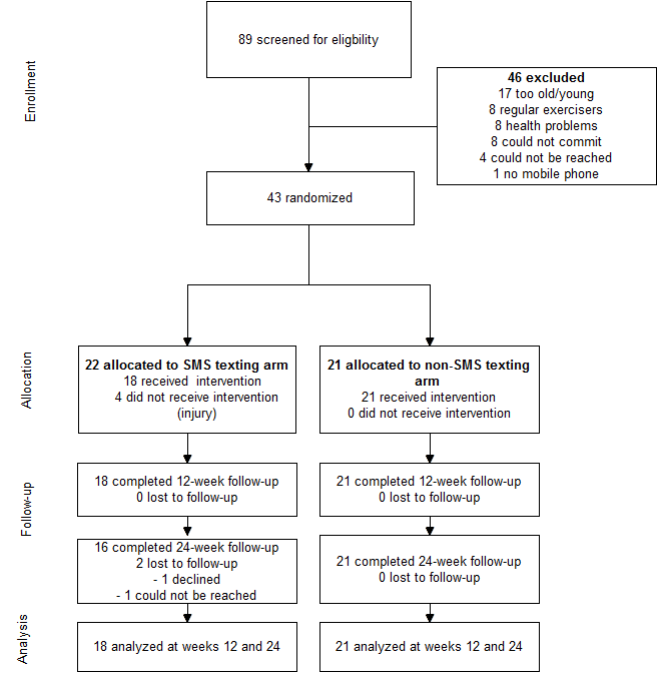
Study participant flow.

Participants were recruited in April and May 2014, and follow-up data were collected until January 2015. Figure 1 depicts the flow of the participants through the study. Of the 89 individuals screened, 43 eligible participants were randomized into the SMS texting arm (n=22) and the non-SMS texting arm (n=21). Table 1 displays the baseline demographic data. Participants had a mean age of 63.3 years (SD 4.5, range 55-70 years). The majority of the participants were female (74%, 32/43), obtained a college or university degree (67%, 29/43), were married (81%, 35/43), not working (77%, 33/43), and reported good health (67%, 29/43). There were no significant differences between the research arms on categorical and continuous variables (*P*>.05). Follow-up assessments at weeks 12 and 24 were completed for 18 (82%, 18/22) SMS texting arm and 21 (100%, 21/21) non-SMS texting arm participants. The overall retention rate was 86% (37/43) from baseline.

**Table 1:** Demographic characteristics of the research participants.

Characteristic		SMS texting (n=22)	Non-SMS texting (n=21)	Total (N=43)
Age (years), mean (SD)		63.64 (4.58)	62.90 (4.48)	63.28 (4.50)
**Sex, n (%)**				
	Male	6 (27)	5 (24)	11 (26)
	Female	16 (73)	16 (76)	32 (74)
**Highest education, n (%)**				
	Secondary	6 (27)	4 (19)	10 (23)
	Postsecondary	1 (5)	3 (14)	4 (9)
	College/university	15 (68)	14 (67)	29 (68)
**Employment status, n (%)**				
	Working	6 (27)	4 (19)	10 (23)
	Not working	16 (73)	17 (81)	33 (77)
**Health status, n (%)**				
	Fair	4 (18)	3 (14)	7 (16)
	Good	16 (73)	13 (62)	29 (67)
	Very good or Excellent	2 (9)	5 (24)	7 (16)
**Marital status, n (%)**				
	Single/Separated/Widowed	4 (18)	4 (19)	8 (19)
	Married	18 (82)	17 (81)	35 (81)

### Outcome Assessments

#### Weekly Exercise Frequency

Over the 12-week intervention period, participants in the SMS texting arm exercised more frequently per week (mean 3.74, SD 1.34) compared to participants in the non-SMS texting arm (mean 2.52, SD 1.85). This difference (mean difference 1.21, bias-corrected and accelerated [BCa] 95% CI 0.18-2.24) was significant (*t*_37_=2.30, *P*=.03, *d*=0.76). The effect of the SMS text messages on weekly exercise frequency was stronger after adjusting for baseline exercise self-efficacy with ANCOVA (*F*_1,36_=6.81, *P*=.01).

Weekly exercise frequency decreased by 0.43 sessions (95% CI 0.12-0.74) from week 12 to week 24 in the overall sample (*F*_1,37_=7.94, *P*=.008). There was no significant research arm by time interaction on weekly exercise frequency (*F*_1,37_=2.46, *P*=.13). However, simple effects analysis revealed a significant decrease of 0.68 sessions within the SMS texting arm (*F*_1,37_=8.93, *P*=.005), whereas no significant decrease was observed in the non-SMS texting arm (*F*_1,37_ =0.85, *P*=.36). Further, an independent *t* test revealed that the SMS texting arm participants did not exercise more frequently (mean 3.07, SD 1.32) than the non-SMS texting arm participants (mean 2.33, SD 1.92) at week 24. The difference between the 2 arms (mean difference 0.74, BCa 95% CI –0.30 to 1.76) was not significant at week 24 (*t*_37_=1.37, *P*=.18, *d*=0.45).

#### Secondary Outcomes

Table 2 lists the effects of the SMS text messages on exercise self-efficacy, PA-related energy expenditure, daily sitting hours, BMI, grip strength, and lower body strength adjusted for the baseline values. There were no significant main or interaction effects (*P*>.05).

**Table 2:** Treatment effects on secondary outcomes.

Outcome	SMS texting, mean (SD) (n=18)			Non-SMS texting, mean (SD) (n=21)			Week 12		Week 24	
	Baseline	Change to week 12^a^	Change to week 24^a^	Baseline	Change to week 12^a^	Change to week 24^a^	Adj difference^a^ (95% CI)	*P*	Adj difference^a^ (95% CI)	P
Exercise self-efficacy score	81.94 (18.74)	–6.47 (19.67)	–3.02 (25.52)	81.55 (17.53)	–9.87 (19.65)	–14.32 (25.51)	–3.39 (–16.21, 9.43)	.60	–11.31 (–27.95, 5.34)	.18
PA-related energy expenditure (weekly MET-minutes)	662.29 (497.29)	383.43 (843.42)	434.69 (728.69)	968.71 (1479.10)	377.43 (842.56)	171.97 (728.22)	–5.99 (–558.14, 546.15)	.98	–262.72 (–739.77, 214.33)	.27
Daily sitting time (hours)	7.28 (3.39)	–0.36 (2.09)	–0.10 (1.90)	8.52 (2.20)	–1.17 (2.09)	–0.77 (1.89)	–0.81 (–2.19, 0.58)	.25	–0.67 (–1.92, 0.58)	.29
BMI (kg/m^2^)	23.50 (3.47)	0.23 (0.51)	0.15 (0.68)	22.39 (2.81)	0.32 (0.51)	0.28 (0.68)	0.09 (–0.25, 0.42)	.60	0.13 (–0.32, 0.58)	.56
Grip strength (kg)	25.93 (8.70)	0.32 (2.01)	1.44 (2.55)	25.51 (6.34)	0.31 (2.01)	1.09 (2.55)	0.02 (–1.32, 1.29)	.98	–0.34 (–2.01, 1.32)	.68
Lower body strength (repetitions in 30-sec chair-stand test)	13.44 (3.42)	2.21 (3.40)	3.55 (2.80)	14.90 (3.81)	2.77 (3.39)	3.43 (2.79)	0.56 (–1.67, 2.80)	.61	–0.12 (–1.96, 1.72)	.90

^a^ Adjusted for baseline.

Over the 24-week study period, a total of 4 adverse events occurred, all in the SMS texting arm (slipped disk: n=2; shoulder injury: n=1; hospitalization: n=1), none of which resulted directly from the study.

#### Qualitative Data

##### Week 12

The semistructured interviews at week 12 revealed that the participants from both arms were satisfied with the exercise program and faced few or no problems performing the exercises. They also reported that they improved their fitness, their health, and experienced elevated mood.

In the SMS texting arm, 9 participants reported few or no barriers to exercising (50%, 9/18). These participants indicated that the SMS text messages had limited impact and that they would have performed similarly without them. In contrast, 9 SMS texting arm participants (50%, 9/18), experienced a number of personal barriers (eg, laziness/tiredness, lack of motivation) to exercising. Despite these barriers, none of these participants discontinued exercise. They affirmed the value of the SMS text messages, which they described as very important and encouraging. They used words such as “cheering,” “hopeful,” and “inspiring” to express how they perceived the SMS text messages. For example, one participant said, “The text messages gave hope that I can do it.” Four participants perceived the messages as an important push for them to exercise when they felt lazy. Interestingly, 4 participants reported feeling guilty when they received the SMS text messages on those days when they had no intention to exercise. They explained that the SMS text messages made them aware of their commitment and then they scheduled their exercise. Finally, 2 participants also reported that the SMS text messages served as a reminder on busy days.

In the non-SMS texting arm, there were also participants who experienced barriers to exercising (52%, 11/21). Six of these participants did not discontinue exercise, but suggested that an encouraging prompt would have been helpful. The remaining 5 participants exercised very infrequently before they discontinued (after 3 to 8 weeks). Three of them thought that reminders or prompts would have been important to help them continue exercising.

##### Week 24

The SMS texting arm participants who experienced few or no barriers to exercising during weeks 1 to 12 reported that they continued to exercise regularly, although some reported less exercise due to various reasons including traveling and busy schedules. Because the SMS text messages were not important to them from the beginning, they did not miss them.

Participants who experienced barriers to exercising during weeks 1 to 12 noted a decrease in barriers and 3 participants reported that the SMS text messages were no longer necessary. However, 2 participants said that it was very difficult for them to continue exercising without the SMS text messages. They reduced their exercise frequency: “I slowed down a little bit without it [text messages] because I did not get reminders.” One participant reported that, without the SMS text messages, she exercised much less because she “did not feel the push and pressure from the text messages.” In the non-SMS texting arm, participants reported few or no changes during weeks 13 to 24 versus weeks 1 to 12.

##### Text Massages Exposure, Content, and Frequency

All SMS text messages were delivered to the SMS texting arm participants as scheduled, without technical problems. Participants who experienced barriers to exercising in the SMS texting arm (50%, 9/18) read all 60 SMS text messages and one participant saved them as well. In comparison, most participants who experienced few or no barriers to exercising ignored the SMS text messages after some time (78%, 7/9). The content of the SMS text messages was perceived as positive. Participants liked the encouragement the SMS text messages provided. One participant said that he “felt that his efforts were appreciated.” Thirteen participants thought that the SMS text messaging frequency was too high (5 text messages per week), whereas 5 participants were in favor of the frequency. Some participants suggested sending more SMS text messages during the initial weeks and reducing the SMS text message frequency over time.

## Discussion

### Principal Results

This is the first RCT investigating a mHealth approach to promote exercise in older adults from a non-HIC. From the results, participants who received 60 encouraging SMS text messages over 12 weeks exercised significantly more than participants who did not receive such SMS text messages (mean difference 1.2 times per week). The SMS text messages were perceived as positive encouragement, especially for participants who experienced a number of barriers to exercising. Exercise frequency decreased significantly in the SMS texting arm when SMS text messages ceased. These findings suggest that SMS text messages have a strong impact on exercise participation in older adults, but the effect does not seem to be sustainable once they are removed.

### Short-Term Effect of the Text Messages

In accordance with previous studies, we found that our SMS text messages had a marked short-term effect on exercise [32-36,42]. A number of reasons why SMS text messaging is effective in behavioral health interventions, particularly among older adults can be suggested. First, SMS text messaging is an easy-to-use mobile phone feature and older adults face fewer barriers to using this technology. This is a great advantage compared with more intricate features and technologies that tend to overwhelm them [28,31]. Our interview results reinforced this explanation because none of the participants experienced difficulties retrieving the SMS text messages. Second, SMS text messaging is generally perceived as a personal way to communicate [33]. This social component might be especially important to older adults, who might perceive a lack of personal interaction when using modern communication technology [28,29,62]. As a result, the attention paid to the intervention content is likely to be high and, in turn, affects intervention effectiveness. Third, some participants mentioned that they benefited from the SMS text messages because it reminded them to exercise.

Additionally, the call for designing behavioral mHealth interventions around effective BCTs has recently increased [63,64]. These BCTs are intended to actively and directly affect behavior [48]. The content of the SMS text messages in the current study was informed by the findings of a meta-analysis that identified the most effective BCTs for promoting exercise self-efficacy and exercise behavior [49]. The BCTs providing instructions to exercise and reinforcing effort of participants to exercise were perceived by a number of participants as influencing their behavior; thus, confirming the relevance of these BCTs.

Finally, our interview analysis revealed that the SMS text messages had a particularly strong impact on participants who experienced a number of barriers to exercise. This is an interesting finding that might explain why the SMS texting arm participants, on average, exercised more compared to the non-SMS texting arm participants. In each research arm, an equal proportion of participants experienced exercise barriers. However, participants who received SMS text messages continued to exercise, whereas a number of participants who did not receive SMS text messages discontinued exercise after some time. Researchers should examine the impact of SMS text messages in older adults who face exercise barriers.

### Long-Term Effect of the Text Messages

Examining the long-term effects of a behavioral health intervention beyond its conclusion is important for research translation [65]. The results of our study indicate that the effect of the SMS text messages was not maintained when the SMS text messages ceased: after 24 weeks there was no difference between the SMS texting and non-SMS texting arms in exercise frequency. Similar findings were reported by Fjeldsoe et al [34], who implemented a 12-week SMS text-messaging intervention.

It is possible that the SMS text messages were not sent long enough to stabilize the acquired exercise routine [65]. Longer interventions might be especially important for older adults who have behavioral patterns that are well established and difficult to change [66]. However, instead of intervening longer with the same intensity, some researchers have also suggested sending prompts in the form of booster text messages to provide occasional support [34,67]. Our interview results do not provide a conclusive picture of the most appropriate behavioral maintenance method. Most participants suggested that the SMS text messages were unnecessary after the 12 weeks and only a few participants indicated reducing their exercise frequency in the absence of the SMS text messages. More research is needed to discern if booster SMS text messages can be an effective means to behavioral maintenance.

Finally, Fjeldsoe et al [65] in their systematic review found interventions with increased face-to-face contact led to more sustainable behavior change. This might be particularly relevant when working with older adults who value personal contact more than other age groups [29]. However, increased personal contact will also lead to increased costs and reduced outreach, decreasing some of the inherent advantages of SMS text messaging, especially when scaled up [67].

### Secondary Outcomes

We did not observe any significant changes on secondary outcomes throughout the course of the study. That exercise self-efficacy did not change in the SMS texting arm compared to the non-SMS texting arm was particularly surprising considering that the BCTs incorporated in the SMS text message content were supposed to promote exercise self-efficacy [49]. One explanation for this is that exercise self-efficacy levels were already high at baseline (82/100) and increments were less likely to occur. McAuley et al [53] suggested measuring exercise self-efficacy approximately 3 weeks after the start of an exercise intervention because participants tend to be too optimistic at baseline and cannot accurately estimate how much effort it might take them to exercise. Measuring exercise self-efficacy after intervention exposure likely leads to a more accurate representation of baseline exercise self-efficacy levels that can serve as a reference for future assessments.

### Limitations and Strengths

This study was limited by a lack of statistical power and the small sample size. Although our sample size calculation was based on the available literature [34,42], we did not expect that the standard deviations of the primary outcome would be as great as we observed. With this, the statistical power was less than the desired 80%. Dropout occurred only in the SMS texting arm. Four participants experienced a study-unrelated injury after a few weeks in the trial and could not continue exercising. We had conducted a rigorous randomization procedure leading to balanced research arms and this pattern of dropouts was unexpected. In this study, we obtained our primary outcome data with an exercise log. The bias of self-reporting is well documented in the literature [68], but using a log for data collection was most appropriate in our study. To ensure data accuracy and validity, participants practiced the data entry at baseline; during the interviews, they did not report any problems filling in the log.

A major strength of the current study was the investigation of behavioral change maintenance in older adults after the SMS text messages were removed; thereby, we filled an important gap in the evidence [42]. We also contribute to the growing body of literature on mHealth interventions in older adults who are less likely to be recruited into such interventions [69]. Further, the current study provides urgently needed evidence that shows that SMS text messaging is potentially effective in promoting health behavior in less developed regions where mobile phone proliferation is highest [37,38,70].

### Conclusions

One of the great potentials of mHealth is that it can reach those most in need of health interventions, including people in non-HICs [38]. However, most knowledge about such interventions is generated in HICs and evidence from other regions is scarce [25,70]. In this study, older Malaysians exposed to SMS text messaging exercised more than those who did not receive SMS text messages, thus demonstrating the effectiveness of such an approach in a non-HIC. The effect of the SMS text messages was not maintained when they were removed, thus indicating a need for research on measures to increase sustainability.

## Appendix

Multimedia Appendix 1 Consort-EHealth checklist V1.6.1.

Multimedia Appendix 2 Screenshots of the online tool used to send SMS text messages to SMS texting arm participants.

Multimedia Appendix 3 Questions from the semistructured interviews (focus on the SMS text messages).

Multimedia Appendix 4 Per protocol analysis.

## Abbreviations

BCa: bias-corrected and accelerated bootstrap
BCT: behavior change technique
EXSE: Exercise Self-Efficacy Scale
HIC: high-income country
IPAQ: International Physical Activity Questionnaire
MET: Metabolic Equivalent of Task
myPAtHS: Malaysian Physical Activity for Health Study
NCD: noncommunicable disease
PA: physical activity
RCT: randomized controlled trial

## References

[1] Rowe JW (2015). Successful aging of societies. Daedalus.

[2] World Health Organization (2015). World Report on Ageing and Health.

[3] United Nations, Department of Economic and Social Affairs Population Division (2013). World Population Ageing 2013.

[4] World Health Organization (2014). World Health Statistics 2014.

[5] Tey NP, Siraj SB, Kamaruzzaman SB, Chin AV, Tan MP, Sinnappan GS, Müller AM (2015). Aging in multi-ethnic Malaysia. Gerontologist.

[6] Department of Statistics Malaysia (2012). Population Projections, Malaysia, 2010-2040.

[7] Christensen K, Doblhammer G, Rau R, Vaupel JW (2009). Ageing populations: The challenges ahead. Lancet.

[8] Rechel B, Grundy E, Robine J, Cylus J, Mackenbach JP, Knai C, McKee M (2013). Ageing in the European Union. Lancet.

[9] Ziegelmann Jochen P, Knoll Nina (2015). Future directions in the study of health behavior among older adults. Gerontology.

[10] Sargent-Cox KA, Butterworth P, Anstey KJ (2015). Role of physical activity in the relationship between mastery and functional health. Gerontologist.

[11] Macera CA, Cavanaugh A, Bellettiere J (2015). State of the art review: Physical activity and older adults. Am J Lifestyle Med.

[12] Erickson KI, Gildengers AG, Butters MA (2013). Physical activity and brain plasticity in late adulthood. Dialogues Clin Neurosci.

[13] Seco J, Abecia LC, Echevarría E, Barbero I, Torres-Unda J, Rodriguez V, Calvo JI (2013). A long-term physical activity training program increases strength and flexibility, and improves balance in older adults. Rehabil Nurs.

[14] Petrella RJ, Lattanzio CN, Demeray A, Varallo V, Blore R (2005). Can adoption of regular exercise later in life prevent metabolic risk for cardiovascular disease?. Diabetes Care.

[15] Chodzko-Zajko WJ, Proctor DN, Fiatarone SM, Minson CT, Nigg CR, Salem GJ, Skinner JS, American College of Sports Medicine (2009). American College of Sports Medicine position stand. Exercise and physical activity for older adults. Med Sci Sports Exerc.

[16] Sun F, Norman IJ, While AE (2013). Physical activity in older people: A systematic review. BMC Public Health.

[17] Macniven R, Bauman A, Abouzeid M (2012). A review of population-based prevalence studies of physical activity in adults in the Asia-Pacific region. BMC Public Health.

[18] Teh CH, Lim KK, Chan YY, Lim KH, Azahadi O, Hamizatul AA, Ummi NY, Syafinaz MS, Kee CC, Yeo PS, Fadhli Y (2014). The prevalence of physical activity and its associated factors among Malaysian adults: Findings from the National Health and Morbidity Survey 2011. Public Health.

[19] Ibrahim S, Karim NA, Oon NL, Ngah WZ (2013). Perceived physical activity barriers related to body weight status and sociodemographic factors among Malaysian men in Klang Valley. BMC Public Health.

[20] Kaur J, Kaur G, Ho BK, Yao WK, Salleh M, Lim KH (2015). Predictors of physical inactivity among elderly Malaysians: Recommendations for policy planning. Asia Pac J Public Health.

[21] Burke LE, Ma J, Azar KM, Bennett GG, Peterson ED, Zheng Y, Riley W, Stephens J, Shah SH, Suffoletto B, Turan TN, Spring B, Steinberger J, Quinn CC (2015). Current science on consumer use of mobile health for cardiovascular disease prevention: A scientific statement from the American Heart Association. Circulation.

[22] Silva BM, Rodrigues JP, de la Torre DI, López-Coronado M, Saleem K (2015). Mobile-health: A review of current state in 2015. J Biomed Inform.

[23] Okorodudu DE, Bosworth HB, Corsino L (2015). Innovative interventions to promote behavioral change in overweight or obese individuals: A review of the literature. Ann Med.

[24] O'Reilly GA, Spruijt-Metz D (2013). Current mHealth technologies for physical activity assessment and promotion. Am J Prev Med.

[25] Hall AK, Cole-Lewis H, Bernhardt JM (2015). Mobile text messaging for health: A systematic review of reviews. Annu Rev Public Health.

[26] Déglise C, Suggs LS, Odermatt P (2012). Short message service (SMS) applications for disease prevention in developing countries. J Med Internet Res.

[27] (2014). The World in 2014: ICT Facts and Figures.

[28] Gell NM, Rosenberg DE, Demiris G, LaCroix AZ, Patel KV (2015). Patterns of technology use among older adults with and without disabilities. Gerontologist.

[29] Vroman KG, Arthanat S, Lysack C (2015). “Who over 65 is online?” Older adults’ dispositions toward information communication technology. Comput Hum Behav.

[30] Parker SJ, Jessel S, Richardson JE, Reid MC (2013). Older adults are mobile too!Identifying the barriers and facilitators to older adults' use of mHealth for pain management. BMC Geriatr.

[31] Zhou J, Rau PP, Salvendy G (2013). Age-related difference in the use of mobile phones. Univ Access Inf Soc.

[32] Prestwich A, Perugini M, Hurling R (2010). Can implementation intentions and text messages promote brisk walking? A randomized trial. Health Psychol.

[33] Fjeldsoe BS, Miller YD, Marshall AL (2010). MobileMums: A randomized controlled trial of an SMS-based physical activity intervention. Ann Behav Med.

[34] Fjeldsoe BS, Miller YD, Graves N, Barnett AG, Marshall AL (2015). Randomized controlled trial of an improved version of MobileMums, an intervention for increasing physical activity in women with young children. Ann Behav Med.

[35] Gell NM, Wadsworth DD (2015). The use of text messaging to promote physical activity in working women: A randomized controlled trial. J Phys Act Health.

[36] Fukuoka Y, Vittinghoff E, Jong SS, Haskell W (2010). Innovation to motivation--pilot study of a mobile phone intervention to increase physical activity among sedentary women. Prev Med.

[37] Gurman TA, Rubin SE, Roess AA (2012). Effectiveness of mHealth behavior change communication interventions in developing countries: A systematic review of the literature. J Health Commun.

[38] Peiris D, Praveen D, Johnson C, Mogulluru K (2014). Use of mHealth systems and tools for non-communicable diseases in low- and middle-income countries: A systematic review. J Cardiovasc Transl Res.

[39] Bloomfield GS, Vedanthan R, Vasudevan L, Kithei A, Were M, Velazquez EJ (2014). Mobile health for non-communicable diseases in Sub-Saharan Africa: A systematic review of the literature and strategic framework for research. Global Health.

[40] Chib A, van Velthoven MH, Car J (2015). mHealth adoption in low-resource environments: A review of the use of mobile healthcare in developing countries. J Health Commun.

[41] Ramachandran A, Snehalatha C, Ram J, Selvam S, Simon M, Nanditha A, Shetty AS, Godsland IF, Chaturvedi N, Majeed A, Oliver N, Toumazou C, Alberti KG, Johnston DG (2013). Effectiveness of mobile phone messaging in prevention of type 2 diabetes by lifestyle modification in men in India: A prospective, parallel-group, randomised controlled trial. Lancet Diabetes Endocrinol.

[42] Kim BH, Glanz K (2013). Text messaging to motivate walking in older African Americans: A randomized controlled trial. Am J Prev Med.

[43] (2014). Hand Phone Users Survey 2014.

[44] Schulz KF, Altman DG, Moher D (2010). CONSORT 2010 statement: Updated guidelines for reporting parallel group randomized trials. Ann Intern Med.

[45] Eysenbach G (2011). CONSORT-EHEALTH: Improving and standardizing evaluation reports of Web-based and mobile health interventions. J Med Internet Res.

[46] (2014). Noncommunicable Diseases Country Profiles 2014.

[47] Gellert P, Ziegelmann JP, Warner LM, Schwarzer R (2011). Physical activity intervention in older adults: Does a participating partner make a difference?. Eur J Ageing.

[48] Michie S, Ashford S, Sniehotta FF, Dombrowski SU, Bishop A, French DP (2011). A refined taxonomy of behaviour change techniques to help people change their physical activity and healthy eating behaviours: The CALO-RE taxonomy. Psychol Health.

[49] Williams SL, French DP (2011). What are the most effective intervention techniques for changing physical activity self-efficacy and physical activity behaviour--and are they the same?. Health Educ Res.

[50] Head KJ, Noar SM, Iannarino NT, Grant HN (2013). Efficacy of text messaging-based interventions for health promotion: A meta-analysis. Soc Sci Med.

[51] Koeneman MA, Verheijden MW, Chinapaw MJ, Hopman-Rock M (2011). Determinants of physical activity and exercise in healthy older adults: A systematic review. Int J Behav Nutr Phys Act.

[52] McAuley E (1993). Self-efficacy and the maintenance of exercise participation in older adults. J Behav Med.

[53] McAuley E, Mailey EL, Mullen SP, Szabo AN, Wójcicki TR, White SM, Gothe N, Olson EA, Kramer AF (2011). Growth trajectories of exercise self-efficacy in older adults: Influence of measures and initial status. Health Psychol.

[54] McAuley E, Jerome GJ, Elavsky S, Marquez DX, Ramsey SN (2003). Predicting long-term maintenance of physical activity in older adults. Prev Med.

[55] Craig CL, Marshall AL, Sjöström M, Bauman AE, Booth ML, Ainsworth BE, Pratt M, Ekelund U, Yngve A, Sallis JF, Oja P (2003). International physical activity questionnaire: 12-country reliability and validity. Med Sci Sports Exerc.

[56] Roberts HC, Denison HJ, Martin HJ, Patel HP, Syddall H, Cooper C, Sayer AA (2011). A review of the measurement of grip strength in clinical and epidemiological studies: Towards a standardised approach. Age Ageing.

[57] Landi F, Cruz-Jentoft AJ, Liperoti R, Russo A, Giovannini S, Tosato M, Capoluongo E, Bernabei R, Onder G (2013). Sarcopenia and mortality risk in frail older persons aged 80 years and older: Results from ilSIRENTE study. Age Ageing.

[58] Taylor ME, Delbaere K, Lord SR, Mikolaizak AS, Brodaty H, Close JC (2014). Neuropsychological, physical, and functional mobility measures associated with falls in cognitively impaired older adults. J Gerontol A Biol Sci Med Sci.

[59] Jones CJ, Rikli RE, Beam WC (1999). A 30-s chair-stand test as a measure of lower body strength in community-residing older adults. Res Q Exerc Sport.

[60] Shumway-Cook A, Silver IF, LeMier M, York S, Cummings P, Koepsell TD (2007). Effectiveness of a community-based multifactorial intervention on falls and fall risk factors in community-living older adults: A randomized, controlled trial. J Gerontol A Biol Sci Med Sci.

[61] Macfarlane DJ, Chou KL, Cheng YH, Chi I (2006). Validity and normative data for thirty-second chair stand test in elderly community-dwelling Hong Kong Chinese. Am J Hum Biol.

[62] Silveira P, van de Langenberg R, van Het RE, Daniel F, Casati F, de Bruin ED (2013). Tablet-based strength-balance training to motivate and improve adherence to exercise in independently living older people: A phase II preclinical exploratory trial. J Med Internet Res.

[63] Direito A, Jiang Y, Whittaker R, Maddison R (2015). Apps for IMproving FITness and increasing physical activity among young people: The AIMFIT pragmatic randomized controlled trial. J Med Internet Res.

[64] Lyons EJ, Lewis ZH, Mayrsohn BG, Rowland JL (2014). Behavior change techniques implemented in electronic lifestyle activity monitors: A systematic content analysis. J Med Internet Res.

[65] Fjeldsoe B, Neuhaus M, Winkler E, Eakin E (2011). Systematic review of maintenance of behavior change following physical activity and dietary interventions. Health Psychol.

[66] Floegel TA, Giacobbi PR, Dzierzewski JM, Aiken-Morgan AT, Roberts B, McCrae CS, Marsiske M, Buman MP (2015). Intervention markers of physical activity maintenance in older adults. Am J Health Behav.

[67] Buchholz SW, Wilbur J, Ingram D, Fogg L (2013). Physical activity text messaging interventions in adults: A systematic review. Worldviews Evid Based Nurs.

[68] Helmerhorst HJ, Brage S, Warren J, Besson H, Ekelund U (2012). A systematic review of reliability and objective criterion-related validity of physical activity questionnaires. Int J Behav Nutr Phys Act.

[69] Müller AM, Khoo S (2014). Non-face-to-face physical activity interventions in older adults: A systematic review. Int J Behav Nutr Phys Act.

[70] Hall CS, Fottrell E, Wilkinson S, Byass P (2014). Assessing the impact of mHealth interventions in low- and middle-income countries--what has been shown to work?. Glob Health Action.

