# Supplementary material for: Text Messaging for Exercise Promotion in Older Adults From an Upper-Middle-Income Country: Randomized Controlled Trial
Source: J Med Internet Res. 2016 Jan 7;18(1):e5. doi: 10.2196/jmir.5235 (PMC4722227; doi:10.2196/jmir.5235)
Supplement: Multimedia Appendix 2 [file jmir_v18i1e5_app2.pptx]

## Slide 1
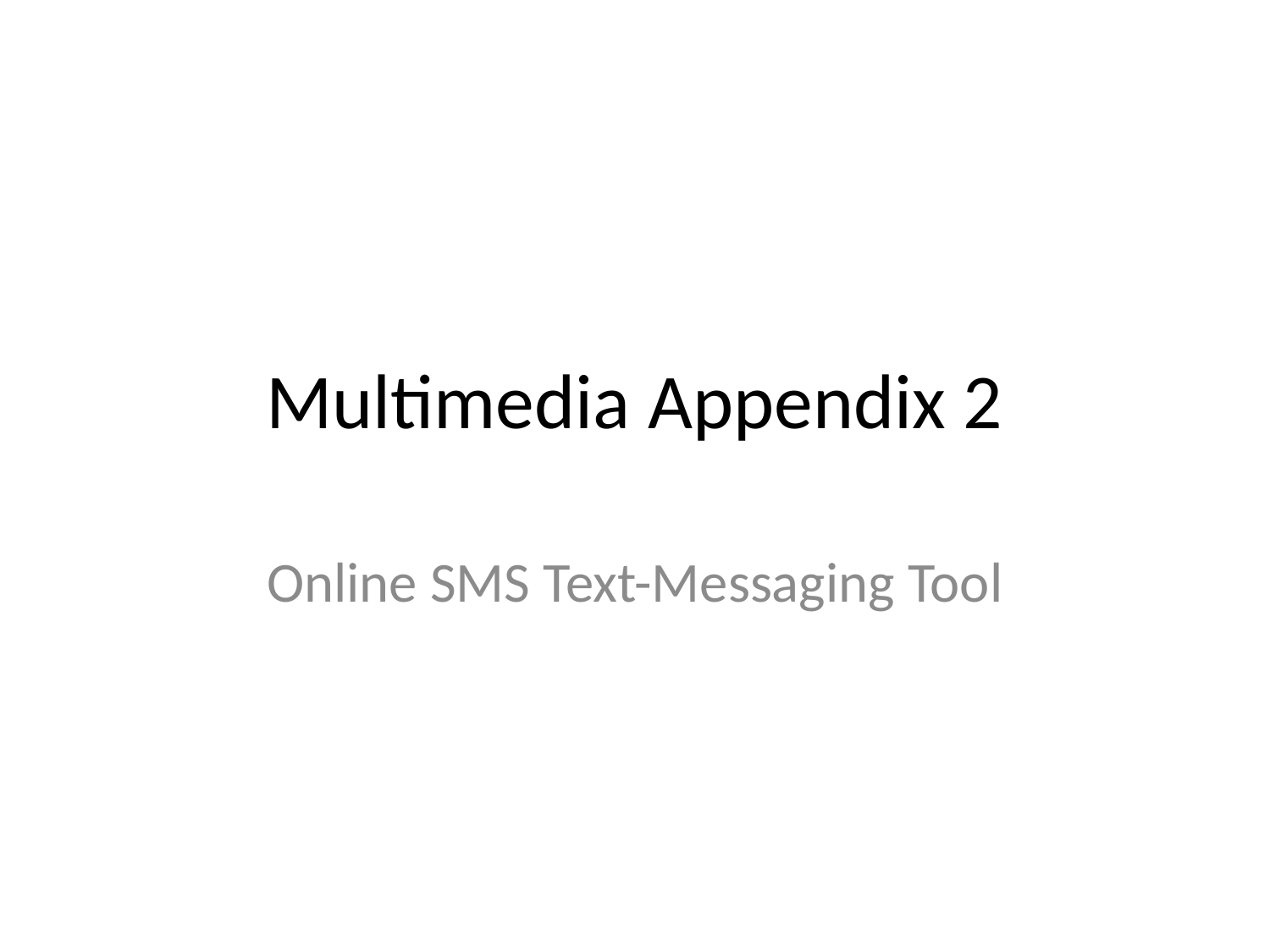

# Multimedia Appendix 2
Online SMS Text-Messaging Tool

## Slide 2
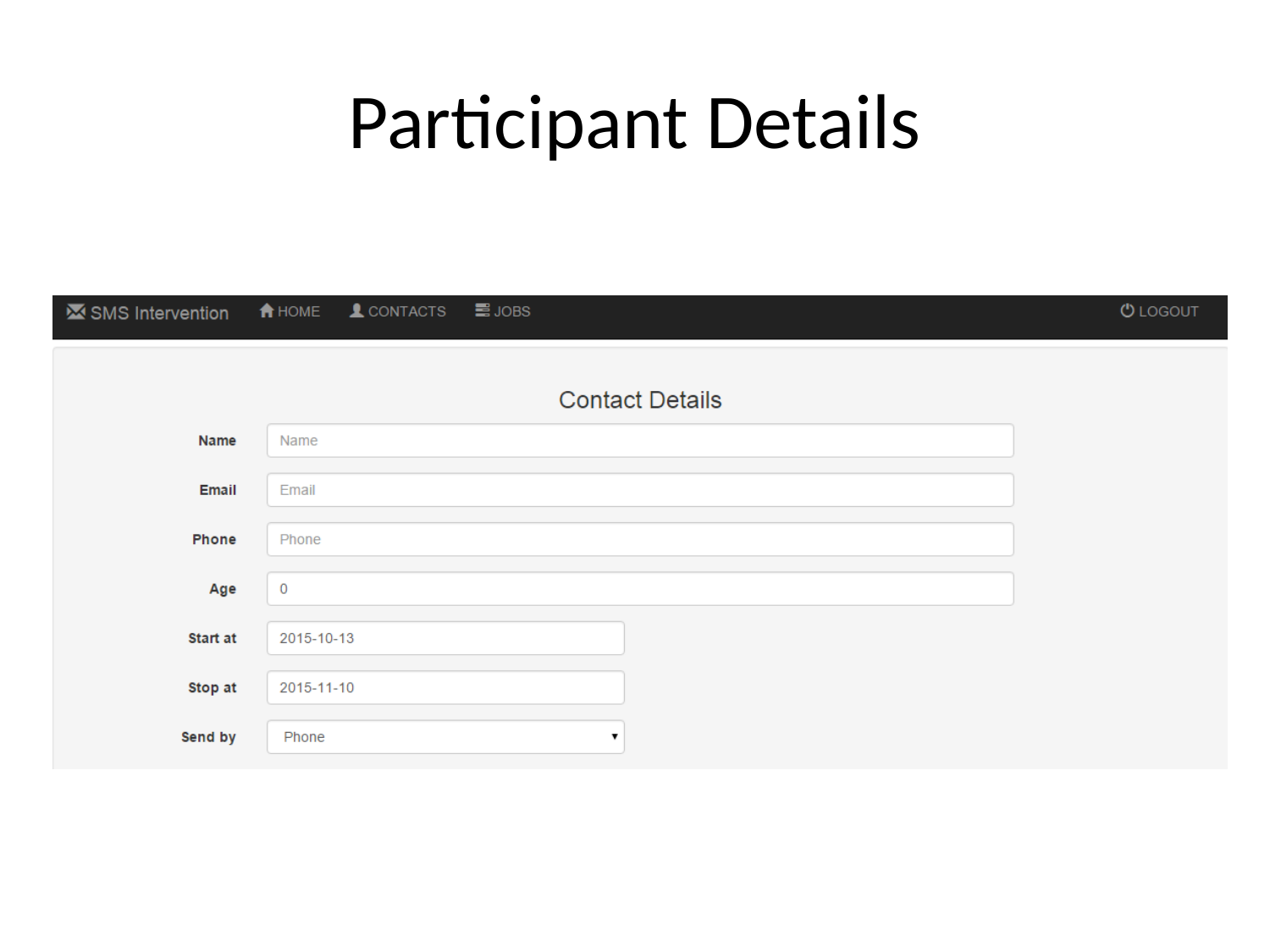

# Participant Details

## Slide 3
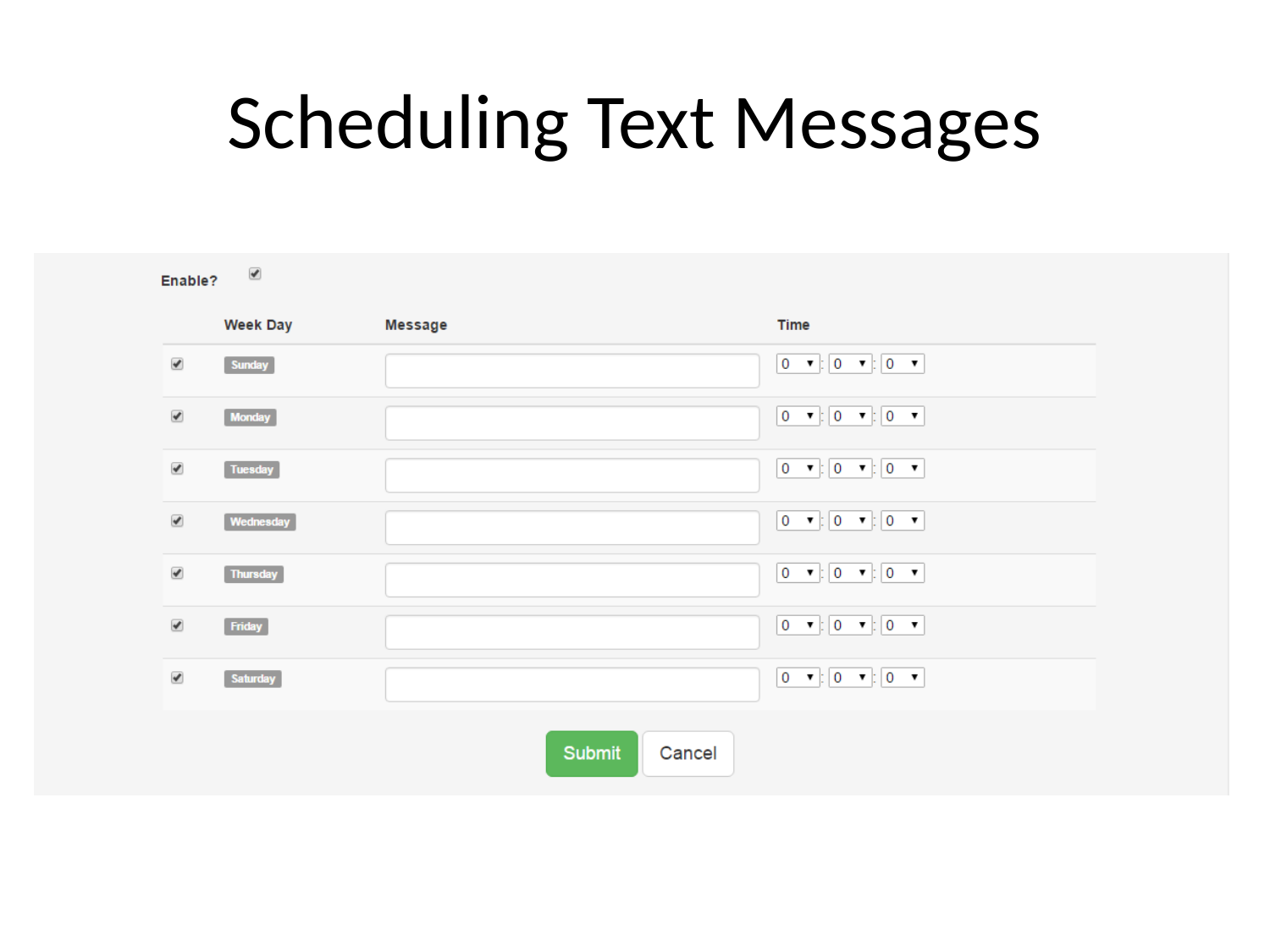

# Scheduling Text Messages

## Slide 4
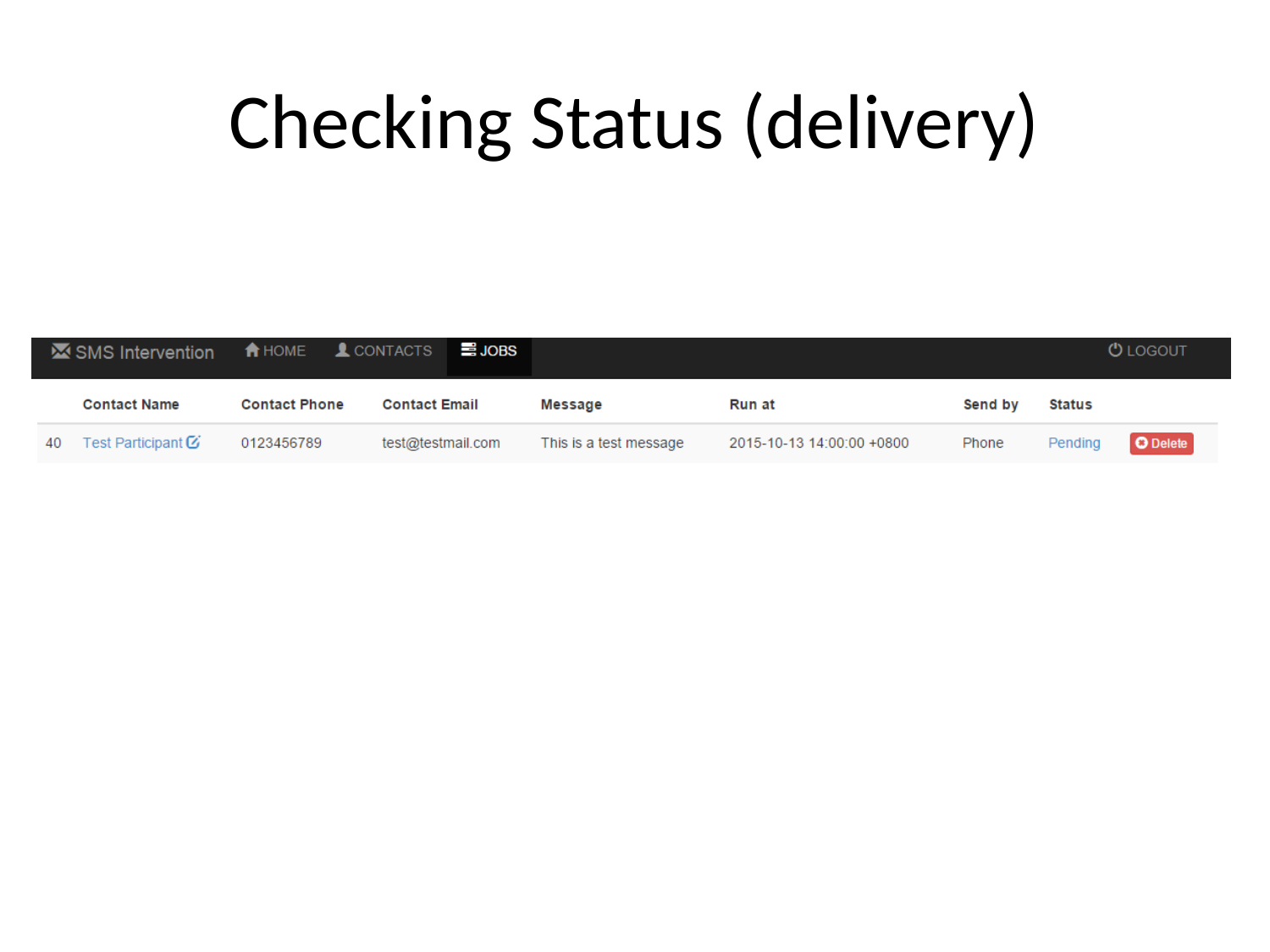

# Checking Status (delivery)
