# Supplementary material for: Text Messaging for Exercise Promotion in Older Adults From an Upper-Middle-Income Country: Randomized Controlled Trial
Source: J Med Internet Res. 2016 Jan 7;18(1):e5. doi: 10.2196/jmir.5235 (PMC4722227; doi:10.2196/jmir.5235)
Supplement: Multimedia Appendix 3 [file jmir_v18i1e5_app3.pdf]

Semi-Structured Interviews (Focus: Text Messages)

Week 12 interview

Introductory Question: “Could you describe your experiences with the $_{\text{my}}$PAtHS exercise program?”

Themes

a) How was your experience with the program (difficulty/ease)?
- Feedback on recording the exercise routine.

b) What do you think about the exercises?
  - Opinion about difficulty/appropriateness
  - Effectiveness (what did you feel?)
  - What would you change?

c) Share some thoughts about the text messages (SMS texting arm only)
- How much (if at all) did the text messages help you to exercise?
- How/Why did it help/not help you?
- What did you mostly do with the text messages?
- What about the content and amount?

d) Recommendations/Suggestions (from participant)

“Is there anything you would like to add?”

Week 24 interview

Introductory Question: “Could you describe your experiences with the $_{\text{my}}$PAtHS exercise program?”

Themes

a) Experiences over the second period
- How easy or difficult was it for you to do it (initially and then after a longer time)?
- Describe changes in how you did the exercise compared to the first period

b) Text messages (only SMS texting arm)
- Could you describe some differences between the time you received text messages and the time you did not receive text messages?
  - Did you miss the text messages?

c) Future
- Final conclusion/feedback about whole study

d) Recommendations/Suggestions

“Is there anything you would like to add?”
