# Supplementary material for: Text Messaging for Exercise Promotion in Older Adults From an Upper-Middle-Income Country: Randomized Controlled Trial
Source: J Med Internet Res. 2016 Jan 7;18(1):e5. doi: 10.2196/jmir.5235 (PMC4722227; doi:10.2196/jmir.5235)
Supplement: Multimedia Appendix 4 [file jmir_v18i1e5_app4.pdf]

Per protocol analysis

Weekly Exercise Frequency

On average, over the 12-week intervention period, the SMS texting arm participants exercised more frequently per week (mean 3.74, SD 1.34) compared to non-SMS texting arm participants (mean 2.52, SD 1.85). This difference (mean difference 1.21, bias-corrected and accelerated [BCa] 95% CI 0.18-2.24) was significant (t_{37}=2.30, *P*=.03 *d*=0.76). The effect of the SMS text messages on weekly exercise frequency was stronger after adjusting for baseline exercise self-efficacy with ANCOVA ($F_{1,36}$=6.81, *P*=.01).

Weekly exercise frequency decreased by 0.47 sessions (95% CI 0.15-0.79) from week 12 to week 24 in the overall sample ($F_{1,35}$=8.74, *P*=.006). There was no significant research arm by time interaction on weekly exercise frequency ($F_{1,35}$=3.09, *P*=.09). However, simple effects analysis revealed a significant decrease of 0.8 sessions within the SMS texting arm ($F_{1,35}$=9.79, *P*=.004), whereas no significant decrease was observed in the non-SMS texting arm ($F_{1,37}$ =0.85, *P*=.36). Further, an independent *t* test revealed that the SMS texting arm participants did not exercise more frequently (mean 2.91, SD 1.09) than the non-SMS texting arm participants (mean 2.33, SD 1.92) at week 24. The difference between the 2 arms (mean difference 0.58, BCa 95% CI –0.35 to 1.55) was not significant at week 24 ($t_{32.7}$=1.16, *P*=.26, *d*=0.39).

Secondary Outcomes

Table 2 lists the effects of the SMS text messages on exercise self-efficacy, PA-related energy expenditure, daily sitting hours, BMI, grip strength, and lower body strength adjusted for the baseline values. There were no significant main or interaction effects (*P*>.05).

Table 2. Treatment effects on secondary outcomes.

Outcome	SMS texting, mean (SD) (n=16)			Non-SMS texting, mean (SD) (n=21)			Week 12		Week 24	
	Baseline	Change to week 12[a]	Change to week 24[a]	Baseline	Change to week 12[a]	Change to week 24[a]	Adjusted difference[a] (95% CI)	*P*	Adjusted difference [a] (95% CI)	P
Exercise self-efficacy score	82.73 (18.48)	–5.62 (20.08)	–1.76 (26.08)	81.55 (17.53)	–9.94 (20.06)	–14.37 (26.06)	–4.32 (–17.86, 9.22)	.51	–12.61 (–30.20, 4.97)	.15
PA-related energy expenditure (weekly MET-minutes)	672.80(528.48)	405.43 (862.04)	461.12 (742.44)	968.71 (1479.10)	367.18 (860.58)	163.22 (742.55)	–38.25 (–621.66, 545.16)	.86	–297.90 (–800.37, 204.57)	.24
Daily sitting time (hours)	7.50 (3.56)	–0.35 (2.16)	–0.03 (1.92)	8.52 (2.20)	–1.21 (2.15)	–0.84 (1.92)	–0.87 (–2.32, 0.59)	.24	–0.81 (–2.11, 0.49)	.21
BMI (kg/m^2)	24.09 (3.20)	0.17 (0.48)	0.07 (0.68)	22.39 (2.81)	0.32 (0.50)	0.29 (0.69)	0.15 (–0.19, 0.49)	.37	0.22 (–0.25, 0.68)	.36
Grip strength (kg)	26.09 (9.04)	0.27 (2.04)	1.53 (2.60)	25.51 (6.34)	0.30 (2.02)	1.08 (2.56)	0.03 (–1.34, 1.39)	.97	–0.45 (–2.19, 1.30)	.61
Lower body strength (repetitions in 30-secchair-stand test)	12.56 (2.40)	2.89 (3.40)	4.31 (2.72)	14.90 (3.81)	2.70 (3.34)	3.43 (2.70)	-0.19 (–2.53, 2.15)	.87	–0.88 (–2.76, 0.99)	.35

[a] Adjusted for baseline
